# Moving the needle for oncology dose optimization: A call for action

**DOI:** 10.1111/cts.13859

**Published:** 2024-06-24

**Authors:** Karthik Venkatakrishnan, Priya Jayachandran, Shirley K. Seo, Piet H. van der Graaf, John A. Wagner, Neeraj Gupta

**Affiliations:** ^1^ EMD Serono Research and Development Institute, Inc. Billerica Massachusetts USA; ^2^ Regeneron Pharmaceuticals, Inc. Tarrytown New York USA; ^3^ Division of Cardiometabolic and Endocrine Pharmacology, Office of Clinical Pharmacology, Office of Translational Sciences, Center for Drug Evaluation and Research U.S. Food and Drug Administration Silver Spring Maryland USA; ^4^ Certara Canterbury UK; ^5^ Leiden University Leiden The Netherlands; ^6^ Koneksa Health New York New York USA; ^7^ Takeda Pharmaceuticals Cambridge Massachusetts USA

Project Optimus is a major FDA initiative aimed at ensuring dose optimization in oncology drug development, moving away from the maximum tolerated dose paradigm and prospectively characterizing dose–response for efficacy and safety for patient‐focused maximization of benefit vs. risk.^1^, ^2^, ^3^ Mitigating toxicities and enhancing the overall benefit vs. risk of oncology therapies necessitates dose optimization with a commitment to evaluation of innovative dosing paradigms including individualized approaches, where appropriate. This requires the quantitative integration of pharmacological mechanisms of action, efficacy, and safety in the context of associated population variability. The problem of dose optimization in the context of the mechanism of action, cancer pathophysiology, and associated population variability sits neatly at the intersection of translational/ precision medicine and quantitative clinical pharmacology and is important to approach with a patient‐focused mindset.

Forums convened on the topic of oncology dose optimization largely engage scientific leaders primarily working on oncology research and development, and cancer medicine. These include workshops organized by Friends of Cancer Research (FOCR),^4^ American Society of Clinical Oncology (ASCO),^5^, ^6^ American Association for Cancer Research (AACR),^7^, ^8^ and the International Society of Pharmacometrics (ISoP)^9^ in partnership with the US Food and Drugs Administration (FDA). Of note, some of these efforts have yielded seminal publications^1^, ^2^, ^10^, ^11^, ^12^, ^13^ and White Papers^14^ offering initial recommendations, including the availability of a Draft FDA guidance on the topic.^15^ We posited that the American Society for Clinical Pharmacology and Therapeutics (ASCPT)—as a premier scientific and professional organization for clinical pharmacology and translational medicine—is optimally positioned to host a discussion of opportunities for our constituent disciplines (e.g., translational science, clinical pharmacology, pharmacometrics) to synergistically address this problem with a multi‐disciplinary approach. To this end, a session was convened at the 2023 ASCPT Annual Meeting bringing together representative scientific leaders from the three scientific journals of the Society—*Clinical Pharmacology and Therapeutics (CPT)*, *Clinical and Translational Science (CTS)*, and *CPT: Pharmacometrics and Systems Pharmacology (PSP)*. These scientific leaders, as at‐large representatives of the disciplines of clinical pharmacology and translational medicine were invited to bring forward their opinions and participate in a fireside chat to identify opportunities for moving the oncology dose optimization needle. This enabled the engagement of a broad group of experts without requiring primary scientific or professional affiliation to the oncology therapeutic area, thereby maximizing the diversity of opinion, out‐of‐the‐box solutions, and fresh perspectives that should help advance us beyond the current state. Ahead of the session at the Annual Meeting, a survey was launched to ASCPT members and meeting attendees to get our finger on the pulse of our Society's membership on issues faced in oncology dose optimization and provide substrate for the fireside chat with the expert panel. Herein, we present the findings from this ASCPT survey and review the insights gained from this Annual Meeting session including recommendations for our scientific communities to join forces and drive progress.

## RESULTS OF ASCPT 2023 SURVEY ON ONCOLOGY DOSE OPTIMIZATION

A focused survey was developed and sent out in February 2023 to meeting attendees and broader ASCPT members on the topic of the session, which consisted of six questions that were relevant to dose optimization (Data S1). The survey was open for 3 weeks and 65 respondents participated in the survey.

We were not only interested in understanding the background of survey respondents that may influence their feedback but also various dose optimization approaches including challenges with various modalities. In response to our question about full‐time engagement with oncology R&D, 58% of respondents were either not engaged or only had part‐time engagement with oncology R&D. This suggested that the survey feedback was from members with diverse backgrounds, as intended. Similarly, we were interested in understanding if strategies for dose optimization in other therapeutic areas are relevant to oncology therapies. And 86% of respondents suggested that strategies from other therapeutic areas are indeed relevant to oncology.

**FIGURE 1:**
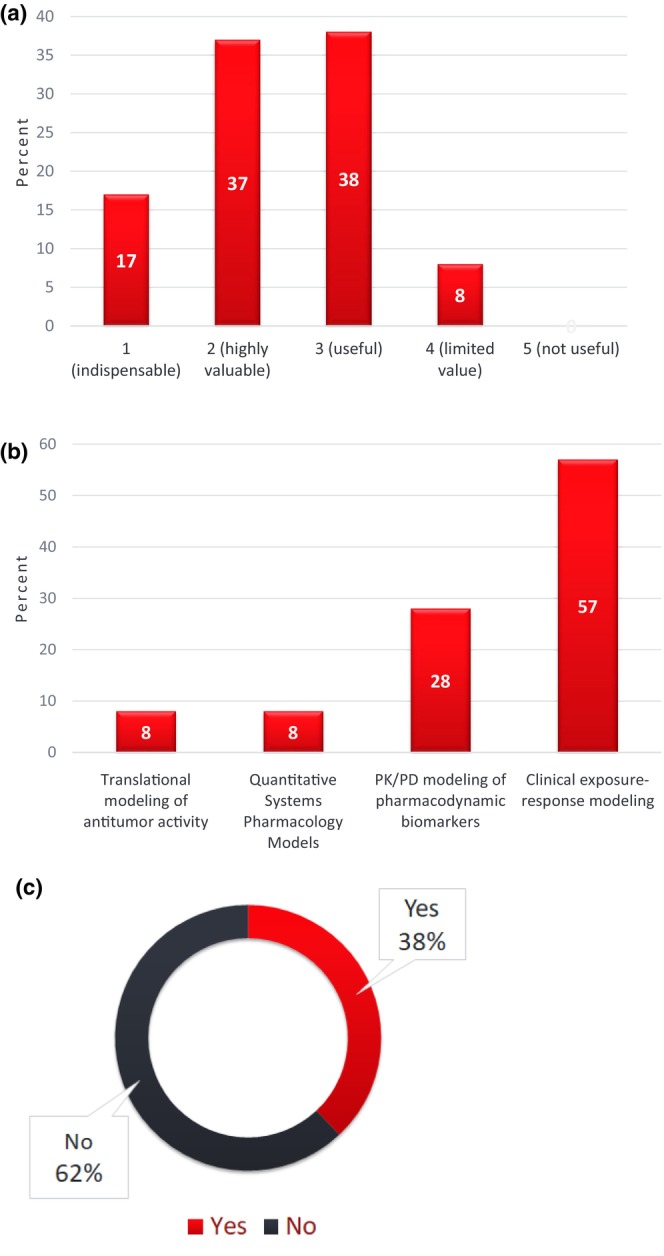
Results of ASCPT 2023 Survey on the value and need for pharmacodynamic biomarkers (panel a), model‐based quantitative approaches (panel b), and randomized dose‐ranging (panel c) for dose optimization in oncology drug development. Survey respondents answered the following questions for the results shown in panels a–c, respectively: (a) On a scale of 1–5, how valuable are pharmacodynamic biomarkers in the overall roadmap for dose optimization in oncology drug development? (b) Which of the following approaches are most relevant and valuable for selecting doses to bring forward in a dose optimization study? (c) Is randomized dose‐ranging evaluation of efficacy and safety an obligate requirement for dose optimization in the clinical development of oncology therapies?

Three questions focused on approaches applied for dose optimization—one on the utility of pharmacodynamic (PD) biomarkers, another one on quantitative approaches for dose selection, and finally a question on study designs for dose optimization with a focus on randomization. And 92% of responses suggest that PD biomarkers are at least useful. Clinical exposure–response modeling (57%) followed by pharmacokinetic (PK)/PD modeling (28%) are the most preferred approaches for selecting doses. Of note, 62% of respondents did not consider randomized dose‐ranging evaluation as necessary for dose optimization, suggesting the value of application on a case‐by‐case approach leveraging the totality of evidence to optimize dose (Figure 1).

**FIGURE 2:**
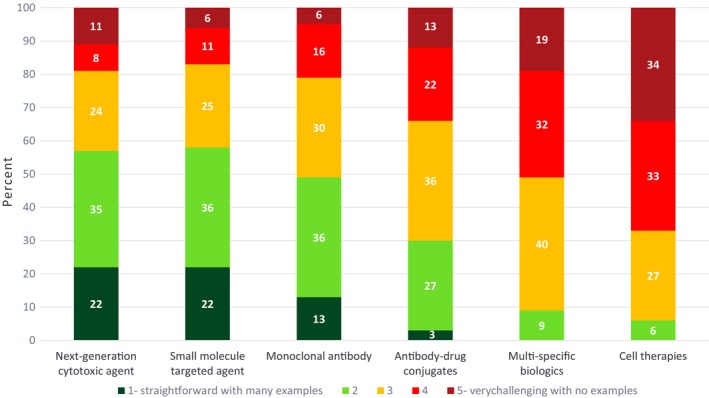
Results of ASCPT 2023 Survey on the level of difficulty in dose optimization in oncology drug development by therapeutic modality. Survey respondents answered the following question: On a scale of 1–5 (1‐ straightforward with many examples to 5‐ very challenging with no examples), how challenging are each of the following modalities with respect to dose optimization in oncology drug development: Next‐generation cytotoxic agent, small molecule targeted agent, monoclonal antibody, antibody–drug conjugate, multi‐specific biologic, cell therapy?

Given that oncology is a therapeutic area with a wide range of modalities from small molecules to cell therapies, we sought to understand the level of challenge associated with dose optimization in developing each of these modalities. Respondents noted that dose optimization for next‐generation cytotoxic agents, small molecule targeted agents, and monoclonal antibodies is relatively straightforward with many historical examples to guide dose selection. However, dose optimization for antibody–drug conjugates was viewed to be moderately complex while newer modalities, such as multi‐specific biologics and cell therapies were considered very challenging with very few or no examples of dose optimization (Figure 2).

## BIOMARKER‐INFORMED TRANSLATIONAL DEVELOPMENT

From a translational perspective, the focus of dose optimization is to find the *right* dose for patients as *swiftly* and *safely* as possible, buttressed by nonclinical and clinical translational data. Translational dose optimization does not always have to be complex. Goldstein et al.^16^ describe a relatively simple concept for translational dose optimization for small molecule targeted oncology agents in the first‐in‐human setting. These suggestions can be implemented today. The approved doses of 25 targeted therapies were examined and the average free concentration at steady state (Css) was determined to be similar to the in vitro cell potency (half‐maximal inhibitory concentration (IC50)). Furthermore, the authors propose a revised first‐in‐human trial design for next‐generation targeted therapy in which dose cohort expansion is initiated at doses less than the maximum tolerated dose when there is evidence of clinical activity and Css exceeds a threshold informed by in vitro cell potency.

Ji et al.^17^ describe another relatively straightforward approach to translational dose optimization in oncology. In this case, the drug is an inhibitor of Porcupine, a membrane‐bound O‐acyltransferase required for Wnt secretion. Wnt pathway is expressed in skin tissues; AXIN2 mRNA expression in skin is a robust and sensitive biomarker for the Wnt pathway. A predominant safety issue in this case is dysgeusia. The authors performed integrated population PK and exposure–response analyses of PD biomarker and safety data to determine the recommended dose for expansion, rather than the conventional maximum tolerated approach.

More complex approaches are also possible and have great utility, particularly for complex therapeutic modalities. Weddell et al.^18^ describe an elegant mechanistic model that characterizes antibody–drug conjugate (ADC) pharmacokinetics and tumor penetration by incorporating tumor growth inhibition via ADC binding radially across solid tumors. The model demonstrates that with low target expression, the potency of the payload should be increased. Furthermore, the model mechanistically links clinical response rates and relapse or resistance to ADC therapies, which could facilitate dose optimization. In another recent example, Susilo et al. leveraged a quantitative systems pharmacology (QSP) model of an anti‐CD20/CD3 T‐cell engaging bispecific antibody, mosunetuzumab, to account for different dosing regimens and inter‐patient heterogeneity in the phase I study to identify biological determinants of clinical response and dose/exposure–response relationships using a novel QSP‐derived digital twins approach.^19^ Approaches of this nature raise opportunities for multidimensional optimization across the dimensions of dose, patient population, and combination partner—a challenge faced routinely in oncology drug development.

The value of new, innovative biomarkers in translational development is continuing to be realized. Recent examples indicate the emerging value of circulating tumor DNA (ctDNA).^20^, ^21^ The translational utility of ctDNA, cancer cell DNA found in the bloodstream, is manifold, including detecting and diagnosing cancer, guiding tumor‐specific treatment, and monitoring treatment and remission. In the context of dose optimization, characterizing the underlying exposure–response relationship for on‐treatment ctDNA dynamics to inform the definition of a clinically active dose range represents an untapped opportunity. Another important innovation has been in the area of digital health technologies such as a proposed multidomain, digital model for capturing functional status and health‐related quality of life in oncology,^22^ which can be particularly relevant to realize the promise of Project Optimus aimed at dosage optimization for improved quality of life during long‐term therapy.

ASCPT, clinical pharmacologists, and translational scientists have a key role in collaboration on dose optimization challenges and opportunities across different stakeholders. ASCPT membership straddles a variety of stakeholders, including academics, industry, regulators, and others to help drive brainstorming and consensus formation. For example, Ji et al.,^23^ in 2018 reported on an ASCPT annual scientific meeting symposium. The authors describe a number of challenges observed before Project Optimus, including postmarket dose‐finding, continued use of traditional 3 + 3 designs, lack of characterization of chronic toxicity, and opportunities for adopting novel designs and testing more than one dose in phase II/III clinical trials. Oncology is one of the most innovative fields in science and yet there are only very few examples of value‐added use of pharmacodynamic biomarkers and dose optimization. Cross‐stakeholder work and Project Optimus are expected to drive the field to increased biomarker‐based and model‐informed solutions for oncology dose‐finding and optimization.

## PATIENT‐FOCUSED DOSE OPTIMIZATION

In their article “The Future of Clinical Trial Design in Oncology,” Spreafico and co‐workers from the Toronto Princess Margaret Cancer Centre^24^ describe how therapeutic approaches in cancer drug discovery and development have shifted from traditional cytotoxic chemotherapy focused on histology‐based targets to molecularly targeted and immune therapies in patient subsets stratified by biomarkers and other diagnostic precision tools. The authors argue that the classical clinical trial paradigm in oncology urgently needs to be transformed to ensure patients will benefit from this scientific revolution in a timely manner. In a wide‐ranging call to action, they present a patient‐centric framework for the next‐generation oncology clinical trials, which maps out the journey of a trial participant as a dynamic and adaptive one continuously leveraging scientific and technological innovations to develop individualized therapeutic strategies. They conclude that “The success of next‐generation clinical trials will be based on the fundamental principles of acting locally to learn globally and treating participants individually to advance the field collectively.” This speaks directly to the opportunity for clinical pharmacology to play a core role in this new paradigm, in particular with regard to dose optimization and individualization based on quantitative, model‐informed approaches that integrate the totality of knowledge and data of the drug, disease, and patient. An example of such an approach is QSP, which in a recent survey conducted by the ISoP was identified as an emerging key tool utilized by oncology drug developers for dose and dose regimen selection and optimization.^25^ A recent example was presented by Li et al.,^26^ who developed a mechanistic model to determine the recommended phase II dose (R2P2D) for epcoritamab, a CD3 × CD20 bispecific antibody (bsAb). The authors justified this novel approach, which integrated preclinical, clinical PK, biomarker, tumor, and response data from the dose‐escalation part of phase I/II trial, on the basis that traditional dose/exposure–response modeling methods may not adequately predict the complex dose/exposure–response relationship for bsAbs. Therefore, trimer formation predicted by the mechanistic model instead of actual clinical measures was used to guide dose prediction.

Along the same lines, in a paper by Chelliah and representatives from a consortium of pharmaceutical companies^27^ the case is made that conventional, empirical pharmacometrics approaches do not fully capitalize on all the available biological and disease knowledge and that QSP models provide a more rational and better alternative to guide complex IO combination therapy development. Their proposal that “virtual patients” simulated by the QSP model under conditions that mimic the actual clinical trial should be added to the drug development paradigm is fully aligned with the earlier‐mentioned call‐to‐action by Spreafico et al.^24^ outlined in *Figure 2* of their publication, suggesting that the future of clinical trial design in oncology may already have arrived.

Poorly characterized dose and schedule may lead to the selection of a dose that provides more toxicity without additional efficacy, severe toxicities that require a high rate of dose reductions, or premature discontinuation and may result in a missed opportunity for continued benefit from the drug. To optimize benefit vs risk with a patient‐focused approach, there remain significant opportunities for model‐based analyses to inform dosing regimen design that may sometimes involve non‐static posology, with the patient response or outcome‐based dose adaptation to ensure individualized dosing for maximizing benefit vs. risk.^28^, ^29^

## PHARMACOSTATISTICAL MODELS AND NOVEL TRIAL DESIGNS

Project Optimus offers a pivotal opportunity to reform the oncology dosing paradigm using a robust quantitative clinical pharmacology framework.^2^, ^3^, ^14^, ^30^, ^31^, ^32^, ^33^ By integrating a model development lifecycle, Bayesian trial designs, and a learning‐and‐confirming mindset across the development spectrum, this framework may be used to prospectively guide dose optimization.

### Model development lifecycle

**FIGURE 3:**
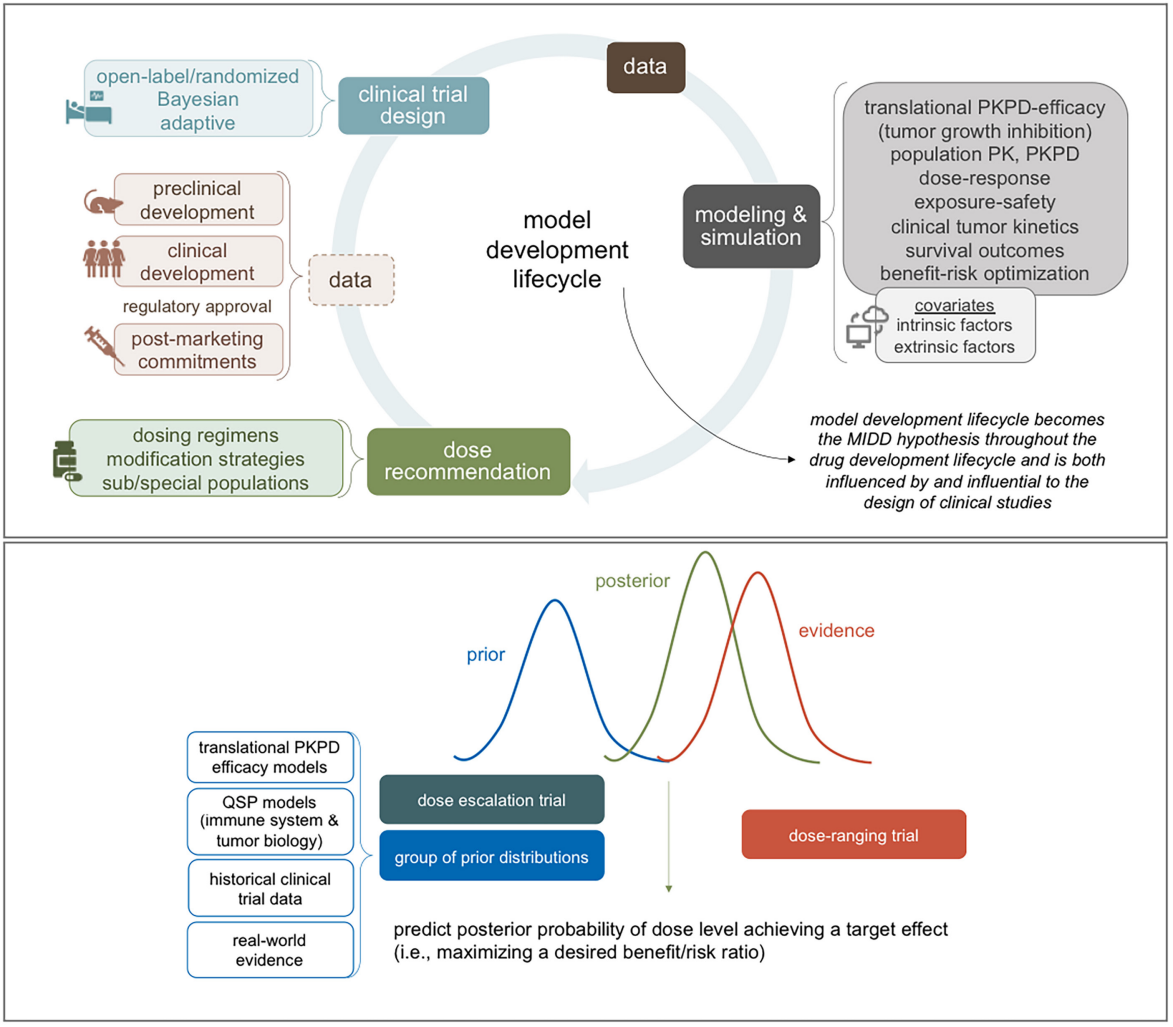
A quantitative clinical pharmacology framework for oncology dose optimization. Model development lifecycle to answer key development questions and guide clinical trial design (top panel) and Bayesian framework to integrate the totality of data from a development program to learn and confirm as evidence is generated (bottom panel). MIDD; Model‐informed drug development; PK, pharmacokinetics; PKPD, pharmacokinetics‐pharmacodynamics; QSP, quantitative systems pharmacology.

The model development lifecycle (Figure 3; top panel) consists of building and revising a collection of models that can be used to answer key development questions that define the drug label. A priori consideration of the quantitative pharmacologic inputs to a model can guide the design elements of a clinical trial such as establishing early data access points of pharmacokinetic and biomarker data within an open‐label design. Bayesian and adaptive components can improve trial efficiency and enable rapid model updates as data emerge for end‐to‐end model development that utilizes the totality of the evidence as it is generated.^34^, ^35^, ^36^ A quantitative framework to predict, interpret, and contextualize emerging data, and sometimes before it is even available through simulations of proposed outcomes, can approximate a real‐time analysis. This model development lifecycle, which is both influenced by and is influential to the design of clinical studies, becomes the model‐informed drug development hypothesis within the drug development lifecycle.

### Innovation in Bayesian clinical trial designs

Contemporary early development trials in oncology have evolved to utilize Bayesian model‐based and model‐assisted designs. They offer seamless movement across early development through expansion cohorts that blend dose escalation with efficacy evaluation.^37^, ^38^ Introducing key optimization metrics like pharmacokinetics and pharmacodynamics can lower the risk of underdosing and integrate key intrinsic and extrinsic factors that explain inter‐individual variability to reduce bias in dose determination. Several recent examples extend the dose‐toxicity design to include exposure to improve the understanding of the benefit–risk relationship for a potential drug.^39^, ^40^, ^41^, ^42^, ^43^, ^44^

### Bayesian framework to inform dose optimization

A learning‐and‐confirming mindset, which is well‐established in drug development, remains under‐utilized in oncology. It can prospectively guide dose optimization by integrating the model development lifecycle and Bayesian trial designs in a Bayesian framework that uses the totality of data from across a development program to learn and confirm as evidence is generated.^36^ Figure 3 (bottom panel) illustrates this framework.

Expanded dose escalation trials that are larger than a similar conventional trial (to overcome the small sample size of early phase trials and heterogeneity in tumor biology and disease that impact the ability to establish early signals of efficacy) can generate robust data to preliminarily characterize the relationships between exposure, toxicity/tolerability, and efficacy. These data and the models developed to describe the data can inform dose selection for a subsequent dose‐ranging trial. The evidence may also be combined in a Bayesian framework with prior models and data, collectively defining a group of prior distributions with some elements being more informative than others based on source and quality. The prior probability distribution and emerging data collected in a dose‐ranging trial can predict the posterior probability of one or more dose levels maximizing a desired benefit–risk ratio. When the quantity and quality of the data and models generated across the early phase trials is high, it can be highly informative to the design of a later phase trial, possibly reducing the trial size and duration so that an effective cancer therapy may become available to patients faster.

### Inter‐disciplinary alliances

Maximizing Bayesian frameworks for dose optimization will depend on inter‐disciplinary alliances between pharmacologists and statisticians,^45^ and the dynamic exchange of ideas and lessons between scientists in industry and regulatory agencies.^5^ The harmonized learnings from these collaborative interactions can further acceptance of the quantitative clinical pharmacology framework and set a precedent for subsequent oncology clinical development programs, ultimately fully realizing the promise of Bayesian methodologies in oncology drug development.

## CROSS‐POLLINATION AND LEARNINGS FROM A NON‐ONCOLOGY PERSPECTIVE

One of the main advantages of examining an oncology challenge as a non‐oncologist is the ability to translate similar principles and successful examples from other therapeutic areas to oncology. These examples can aid in enriching a holistic approach toward solving longstanding problems. One clear correlate is in HIV drug discovery. In the 1980s, the average life expectancy following an AIDS diagnosis was approximately 1 year. And by the early 1990s, HIV was the leading cause of death among Americans aged 25–44. In many ways, much like with cancer, the urgency to save lives and the need for therapeutics to control the epidemic fueled innovation and discovery. The beginning of that discovery phase did lead to some unsophisticated dosing—zidovudine was initially studied and approved at a dosage of 200 mg q4h, which caused severe anemia and neutropenia. However, more fine‐tuning of the dose through clinical trials eventually led to its current dosage regimen of 300 mg twice daily. Several advancements along the way led to HIV infection largely being regarded as a chronic condition with near‐normal life expectancy for patients and a much‐improved quality of life. Some of these advancements included a deeper and continual understanding of the pharmacological mechanisms of antiretroviral agents, the development of enhanced diagnostics, and the acceptance of early biomarkers. When these approaches were deployed simultaneously, the result was a highly integrated, advanced methodology for solving an urgent public health problem. One of the biggest challenges that the area of oncology faces now is the issue of how to operationalize. No matter the disease area, proper prospective dose‐finding at the outset, focusing on a broad strategy, and early biomarker work can be incredibly beneficial. Several examples, such as blood pressure reduction, lowering of HbA1c, and reduction in LDL cholesterol have been studied extensively and correlated so strongly with outcomes of interest that they are all now considered surrogate end points. Therefore, the exploration of biomarkers at an early stage can be an incredibly critical area of investment with the potential for a high rate of return.

## CONCLUDING REMARKS AND FUTURE PERSPECTIVES

**FIGURE 4:**
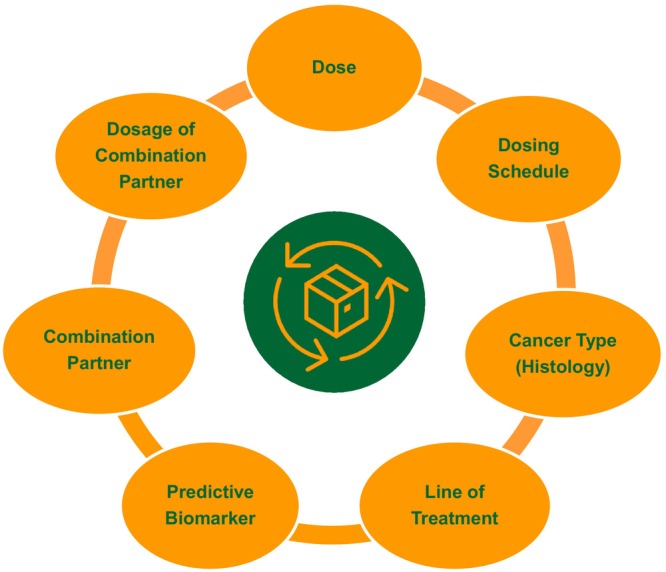
Oncology drug development involves multidimensional optimization where *Dose* is one of several dimensions, demanding inter‐connected evidence generation with a *Totality of Evidence* mindset, leveraging translational, quantitative, and clinical pharmacology approaches in an integrative and iterative manner.

Oncology is a major therapeutic area in pharmaceutical R&D with diverse therapeutic modalities and explosive advances in precision medicine. Drug development in oncology involves multidimensional optimization, where *Dose* is one of several dimensions (Figure 4), demanding inter‐connected and iterative evidence generation with a *Totality of Evidence* mindset. When approaching the development of tailored precision medicines in cancers with diverse molecular footprints, dose selection cannot be approached as a *One Size Fits all* approach. Diversity in tumor molecular profile and host immunophenotype are important considerations in the discovery and development of precision oncology therapies at the right dose and dosing schedule for all patients. Advances in biomarker sciences and translational informatics are enabling deep characterization of the diversity of cancer biology and immunology across patient populations, with rapidly emerging applications of machine learning and artificial intelligence to harness such multimodal multidimensional data. These data represent invaluable inputs for the development of next‐generation QSP platforms and their seamless integration in clinical drug development to identify the biological determinants of variability in clinical response and dosage requirements. Such integrated approaches have the potential to elevate the efficiency and fidelity of our current approaches to patient selection, combination partner selection, and dosage optimization.

As evident from the results of our 2023 ASCPT survey, randomized dose‐ranging evaluation was not considered as an obligate requirement for dose optimization in all cases by about 60% of survey respondents. Indeed, examples exist where the application of biomarker‐based and model‐informed integrative approaches with a *Totality of Evidence* mindset have enabled confidence in the approved dosage of anticancer therapies, with many published success stories.^26^, ^46^, ^47^, ^48^ In a *Totality of Evidence* approach, evidence is substantiated through the confidence gained from consistency across multiple approaches and data sources integrated in a mechanism‐informed manner through modeling and simulation.^49^ Such holistic integrative approaches are critically important when approaching the development of novel therapeutic modalities such as multi‐specific biologics and cell therapies, where our survey indeed suggested that dose optimization will be most challenging. We are pleased to note steady progress in this area, with several recent publications across all three ASCPT Journals highlighting advances in translational, quantitative, and clinical pharmacology applications for these emerging anticancer therapeutics.^50^, ^51^, ^52^, ^53^, ^54^, ^55^ As we learn from present and future real‐life examples and continue to refine best practices in oncology dose optimization, we invite our readership and cross‐sector practitioners to submit these advances for timely publication. We trust that the scientific discussion and rigorous debate that will ensue across our communities of practice, further facilitated by ASCPT's Networks and Communities, will go a long way in elevating patient‐focused evidence generation for maximizing the benefit/risk profile of next‐generation oncology therapies.

## Supporting information


Data S1.
